# Effect of Heat Treatment on Gelatin Properties and the Construction of High Internal Phase Emulsions for 3D Printing

**DOI:** 10.3390/foods13244009

**Published:** 2024-12-11

**Authors:** Yixiao Wang, Ling Zhang, Geng Cao, Zhaorui Li, Ming Du

**Affiliations:** SKL of Marine Food Processing & Safety Control, National Engineering Research Center of Seafood, School of Food Science and Technology, Dalian Polytechnic University, Dalian 116034, China; wangyixiao0816@163.com (Y.W.); bu52@163.com (G.C.); 18165210790@163.com (Z.L.)

**Keywords:** gelatin, heat treatment, HPIEs, 3D printing

## Abstract

The effect of tilapia skin gelatin properties on the characteristics of high internal phase emulsions (HIPEs) and the quality of 3D printing remains unidentified. In this work, HIPEs were constructed by gelatin with various properties that were obtained by heat treatment. The results indicated that the gelatin undergoes degradation gradually with an increase in heating intensity. The highest values of intrinsic fluorescence intensity, surface hydrophobicity, and emulsification were obtained when the heating time was 5 h. The gel strength and hardness of gelatin hydrogels were negatively correlated with heat treatment temperature. HIPEs constructed by gelatin extracted at 70 °C demonstrated a suitable material for 3D printing. The storage modulus (G′) and viscosity of HIPEs exhibited a similar tendency as the gel strength of gelatin. The microstructure of HIPEs revealed that gelatin established a gel network around oil droplets, and the higher G′ of HIPEs corresponded to a more compact network structure. This study elucidated the correlation between the structure and properties of gelatin, offering essential insights for the formulation of HIPEs by natural gelatin, which is suitable for applications across several domains.

## 1. Introduction

Gelatin, derived from the partial degradation of collagen, is a fibrous protein that exhibits various characteristics such as gelation, foaming, low antigenicity, and exceptional biocompatibility [1]. Gelatin with varying properties can be applied in numerous fields, including medicine, materials, and food. The high-gel-strength gelatin was employed as scaffolds or hemostatic sponges in medicine, whereas gelatin with weak gel strength functions as a thickening ingredient for various products or facilitates the production of easily swallowable food for the elderly [2,3]. Gelatin serves as an emulsifier for the building of emulsions. Wang et al. enhanced the interface and antioxidant characteristics of the emulsion by modifying the molecular interactions between gelatin and catechin [4]. Moreover, studies indicated that glycosylation and phosphorylation influence the rheological properties of gelatin emulsions [5,6].

In recent years, the specific needs of various consumers, including athletes, the elderly, and children, have attracted increased attention, leading to the emergence of designed food as a new trend. Three-dimensional printing received major interest due to its simplicity, flexibility, individualized nutritional design, customized nutrition, and simplified supply chain [7,8]. High internal phase emulsions (HIPEs) are emulsions with an oil phase volume percentage above 74%. It is an appealing material with several applications in the culinary industry, including sauces, cream products, fat alternatives, and nutrition delivery systems [8,9]. HIPEs exhibited excellent extrudability due to its superior rheological characteristics, making it a potential material for 3D printing. Nonetheless, maintaining the designed shape was challenging, and phase separation may readily occur during the extrusion process, which limits their applicability in 3D printing.

Prior studies have demonstrated that the stability and 3D printing properties of gelatin emulsion can be improved through the modification of gelatin or its combination with polysaccharides [6,10]. Biological and chemical methods can enhance the precision of gelatin in 3D printing, such as transglutaminase [11] and the Hofmeister effect [12]. Extracting gelatin from tilapia skin is an effective method for waste disposal, pollution reduction, and the generation of value-added products, owing to the fast growth rate, adaptability to various environmental conditions, efficient reproductive ability, safety, and affordability of tilapia. Currently, the impact of tilapia skin gelatin properties on HIPE characteristics and 3D printing have not been thoroughly studied.

In this study, the properties of gelatin were adjusted by varying heat treatment temperatures and times, and the correlation between heat treatment intensity and gelatin properties was investigated. HIPEs were constructed by gelatin with different properties, and a test was conducted on their rheological and 3D print properties. The eco-friendly, affordable, convenient, and unmodified gelatin-based HIPEs exhibited excellent 3D printing performance. These studies provide valuable information for the development of and a specific type of material that are suitable for 3D printing.

## 2. Materials and Methods

### 2.1. Materials and Chemicals

Dried tilapia fish skin was obtained from Hongjiarui Aquatic Flagship Store and stored at room temperature. Soybean oil was purchased from a local supermarket (Dalian, China). Na_2_HPO_4_, NaH_2_PO_4_, HCl, NaOH, Coomassie brilliant blue G-250, and 8-Anilino-1-Naphthalenesulfonic acid were purchased from Aladdin Reagent Co., Ltd. (Shanghai, China). All reagents were analytical grade in this study, and all solutions were prepared in double-distilled water.

### 2.2. Preparation of Gelatin, Gelatin Hydrogels, and High Internal Phase Emulsions (HIPEs)

Gelatin from tilapia skin was extracted as described in a previous work with a slight modification [13]. Tilapia skins were trimmed into 1.0 cm × 1.0 cm pieces and placed in a 0.1 mol/L NaOH solution (skin/solution ratio was 1:6, *w*/*v*) for 2h. Pieces of skin were washed with distilled water until the pH was 7.0 ± 0.2. Then, stirring at 20–25 °C for 1 h, 0.05 M of HCl was mixed with tilapia skin for 1 h (skin/solution ratio was 1:6, *w*/*v*). After that, the skin was washed until the pH became 7.0 ± 0.2 again. The procedure of gelatin extraction was as follows: The ratio of skin-to-water was 1:1 (*w*/*v*). The heating temperatures were 50 °C, 70 °C, and 90 °C, and the heating times were 2 h, 5 h, and 8 h, respectively. The gelatin of 50-2 indicated that the gelatin was obtained after heating at 50 °C for 2 h, and so on. The skin residue was removed by filtration and centrifuged at 10,000× *g* for 20 min. The supernatant was freeze-dried and stored in a dryer.

The tilapia skin gelatin solution (5%, *w*/*v*) was prepared in deionized water and stirred at 50 °C for 30 min using a magnetic stirrer. An ultrasonic cleaner was used to remove air bubbles. Further, the solution of gelatin was transferred to 4 °C for 12 h to prepare gelatin hydrogel. The hydrogel was constructed by gelatin, which was treated at 50 °C for 2 h, called H-50-2, and so on.

HIPEs were prepared by combining 16 mL of soybean oil with 4 mL of gelatin solution (80 mg/mL) at room temperature. An high-speed homogenizer (T25, IKA, Staufen, Germany) was used to emulsify the mixture at 8000 rpm for 90 s. The HIPEs were constructed by gelatin, which was treated at 50 °C for 2 h, called E-50-2, and so on.

### 2.3. Structure and Properties of Gelatins Under Various Heat Treatment

#### 2.3.1. Sodium Dodecyl Sulfate-Polyacrylamide Gel Electrophoresis (SDS-PAGE) of Gelatins

The SDS-PAGE method was based on the method mentioned by Laemmli with a slight modification [14]. A 12.5% separating gel and 3% stacking gel were used, and the constant voltage was 80 V. Gelatins were suspended in double-distilled water by incubating at 50 °C for 30 min (5 mg/mL), mixed with a protein loading buffer (5×) at a ratio of 1:4 (*v*/*v*), and then the solution was was subjected to boiling water for 5 min. Subsequently, 8 μL of the mixture was loaded into the gel tank. Spectra Broad Multicolor Range Protein Ladder Standard (5–245 kDa, Solarbio, Beijing, China) was used as the protein standard. The gel was stained in Coomassie brilliant blue R-250 and de-stained in a solution with acetic acid and methanol. A protein marker was used to analyze the molecular weight of gelatins.

#### 2.3.2. Fourier Transform Spectroscopy (FTIR) of Gelatins

The measurement of Fourier transform spectroscopy (FTIR) was performed as described by Zhao et al. [15]. Two milligrams of freeze-dried gelatin were mixed with 200 mg of potassium bromide (spectrum pure), respectively. The mixture was ground into a powder and then pressed into a disk. The FTIR of gelatins were measured by an infrared spectrophotometer (Frontier, PerkinElmer Frontier, Waltham, MA, USA). The range was recorded from 400 to 4000 cm^−1^ and collected in 64 scans with a resolution of 4 cm^−1^. The pure potassium bromide was used as the background.

#### 2.3.3. Intrinsic Fluorescence Spectroscopy Measurements

The intrinsic fluorescence emission spectra of tilapia skin gelatin were assessedu according to a previous report with some modifications. A spectrometer (F-2700, Hitachi, Tokyo, Japan) was used in this test at 25 °C. Protein solutions (0.5 mg/mL) in a phosphate buffer (10 mmol/L, pH 7.0) were put in a 1 cm × 1 cm quartz cuvette. The photomultiplier tube voltage, excitation wavelength, and excitation and emission slit widths were set as 700 V, 280 nm, 2.5 nm, and 2.5 nm [16].

#### 2.3.4. Determination of Surface Hydrophobicity (H_0_)

Surface hydrophobicity (*H*_0_) was determined by a fluorescence probe method [17]. Gelatin solutions were diluted with a 10 mM sodium phosphate buffer (pH 7.0) to obtain concentrations ranging from 0.02 mg/mL to 1 mg/mL. An amount of 4 mL of gelatin solutions was mixed with 50 mL of the ANS solution (8 × 10^−3^ M in 10 mM of pH 7.0 PBS), respectively, and kept in the dark at room temperature for 15 min. Subsequently, the fluorescence intensity of each solution was measured by a fluorescence spectrophotometer (F-2700, Hitachi, Tokyo, Japan). The excitation wavelengths, emission wavelengths, and slit width were 390 nm, 470 nm, and 5 nm, respectively. The value of *H*_0_ was defined as the slope of fluorescence intensity against protein concentration, and the slope was calculated by the least squares linear regression analysis.

#### 2.3.5. Determination of Emulsifying Properties

The emulsifying activity index (EAI) and emulsifying stability index (ESI) were used with a method previously reported with modifications [18]. Ten mL of a 1% (*w*/*v*) protein solution and ten mL of soybean oil were mixed, and a high-speed homogenizer (Ultra Turrax, T25, IKA, Staufen, Germany) was used to prepare emulsion at 8000 rpm for 90 s. A 20 μL emulsion was taken from the end of a beaker and mixed with 2 mL of SDS (0.1%) at 0 min and 10 min. The absorbance was measured at the wavelength of 500 nm by a spectrophotometer (Lambda 35, PerkinElmer, Waltham, MA, USA), and the blank solution was 0.1% SDS.

(1) $$EAl\left(m^{2}/g\right)=\frac{2\times 2.303\times A_{0}\times N}{\left[\Phi \times C\;\times 10000\right]}$$

(2) $$ESI\left(min\right)=A_{0}\times △t/△A$$

where A_0_ is the absorbance at 0 min, A_10_ is the absorbance at 10 min, N is dilution multiple 11, Φ is the volume fraction of oil phase 0.5, C is the protein concentration g/mL, Δt = 10 min, and ΔA = A_0_ − A_10_.

### 2.4. Texture Determination of Gelatin Hydrogels

The texture characteristics of gelatin hydrogels were mesusured according to a previous study [19]. A spherical probe (P/5s) and a cylindrical probe (P50) were used to determine the gel strength and TPA of gelatin hydrogels by a TA. Plus texture analyzer (Stable Micro Systems Ltd., Godalming, UK). The rigger type, strain, pre-test speed, test speed and post-test speed were set as follows: 5 g, 40%, 2.0 mm/s, 5.0 mm/s and 5.0 mm/s.

### 2.5. Practical 3D Printing of HIPEs

The method of 3D printing using HIPEs was performed following a report conducted by Du et al. [8]. A 3D printer (FPE2, Fochif Mechatronics Technology Co., Ltd., Shanghai, China) was used to print the model of Chinese knots. The extrusion parameters were set as follows: the inner nozzle diameter was 1 mm, and the extrusion rate was 1 mm/s. The images of HIPEs were recorded.

### 2.6. Rheological Analysis s of HIPEs

The rheology properties of HIPEs were evaluated using a Discovery hR-1 Rheometer (TA, New Castle, DE, USA), as reported by Chen at et al. [20].

A parallel plate measuring 40 mm in diameter was used in this study, and the gap was 1.00 mm. Amplitude sweeps were conducted with a strain range of 0.1% to 1000% (frequency was 1 Hz, temperature was 25 °C) to determine the linear viscoelastic region (LVER) of HIPEs.

Frequency sweeps (1–10 Hz, strain was 1%) of HIPEs were performed to obtain the storage modulus (G′) and loss modulus (G″).

The 3-ITT contained a three-step shear rate test. The initial stage involved a shearing rate of 0.1 s^−1^ for 200 s. The subsequent stage required subjecting the HIPEs to a shearing rate of 10 s^−1^ for 200 s. Finally, HIPEs were subjected to a shearing rate of 0.1 s^−1^ for 200 s again. The recovery rate (*RR*) was determined by the following formula [21]:

(3) $$RR\%=\frac{\eta_{200}}{\eta_{600}}\times 100$$

where $\eta_{200}$ is the viscosity value of the EGs at 200 s, and $\eta_{600}$ represents the viscosity value of the EGs at 600 s.

The HIPEs underwent shear at ascending (AS) shear rates from 10 to 80 s^−1^, followed by shear at descending (DS) shear rates from 80 to 10 s^−1^. The thixotropic index (Ti) and recovery index (Ri%) were calculated by Equations (4) and (5).

(4) $$Ti=\frac{\eta_{\gamma 10}}{\eta_{\gamma 80}}\left(\mathrm{AS}↑\right)$$

where Ti% is Thixotropic index, $\eta_{\gamma 10}$ is the viscosity at shear rate 10 s^−1^, and $\eta_{\gamma 80}$ is the viscosity at shear rate 80 s^−1^.

(5) $$Ri\;\%=\frac{\eta_{\gamma '10}}{\eta_{\gamma 10}}\times 100\left(\mathrm{AS}↑\;\to \;\mathrm{DS}↓\right)$$

where Ri% is recovery index, $\eta_{\gamma '10}$ is the viscosity at ascending shear rate 10 s^−1^, and $\eta_{\gamma 10}$ is the viscosity at descending shear rate 10 s^−1^, respectively.

### 2.7. Microstructure Observation of HIPEs

A cold field emission scanning electron microscope (cryo-SEM) (SU8010, Hitachi, Japan) was applied to examine the microstructural characterization of HIPEs. HIPEs were cut into appropriate pieces before being loaded onto the plate. Then, liquid nitrogen was used to freeze samples and preserve the microstructures of HIPEs. The samples were then cut to the suitable height, sublimated for 30 min at −90 °C, and sprayed with gold for 60 s. The microstructure of HIPEs was observed at 10 kV [22].

### 2.8. Statistical Analysis

All experiments were repeated three times, and data were analyzed using SPSS 26.0 software (SPSS Inc. Chicago, IL, USA). The method of multiple comparisons of mean values and variance (ANOVA) was performed, followed by Duncan’s multiple range test. The values of *p* less than 0.05 were considered significant differences.

## 3. Results and Discussion

### 3.1. Structure and Properties of Gelatins Under Various Heat Treatment Conditions

#### 3.1.1. Molecular Weight Distribution of Tilapia Skin Gelatins

**Figure 1:**
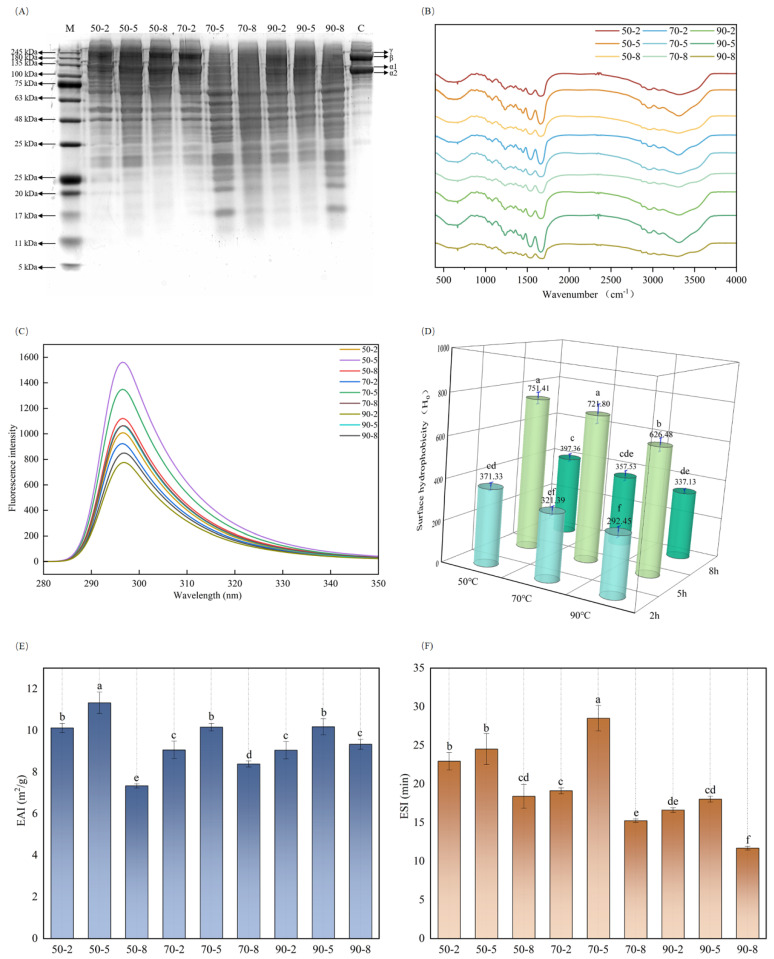
Structure and properties of gelatins extracted at different temperatures and times. (**A**) Sodium dodecyl sulfate-polyacrylamide gel electrophoresis (SDS-PAGE), (**B**) Fourier transform spectroscopy (FTIR), (**C**) Intrinsic fluorescence spectroscopy measurements, (**D**) Surface hydrophobicity (H_0_), (**E**) Emulsifying activity index (EAI), (**F**) Emulsifying stability index (ESI). Note: Different letters indicate the significant difference between samples (*p* < 0.05).

Studies indicated that alterations in molecular weight distribution might influence the hydrophobicity [23], emulsification [24], and gelation of protein. Figure 1A shows the patterns of gelatin treated by various heat treatments and collagen. The presence of two chains, α1 and α2, with a molecular weight of roughly 135 kDa, was discovered, indicating the integrity of Tilapia skin collagen (C). The intensity of the α1 chain was found to be approximately two-fold higher than the α2 chain, suggesting that the skin collagen of tilapia was mainly composed of type I [25]. Further, the bands observed at 245 kDa and 180 kDa correspond to the presence of γ and β chains, respectively. Upon the heating temperature of 50 °C, α1, α2, β and γ bands were observed. The hydrolysis of tilapia skin gelatin was found to be slight, and the results obtained under 2 h, 5 h, and 8 h were similar. The pattern intensity of α, β, and γ decreased obviously when the condition of the heat treatment was 70 °C for 5 h, resulting in an increase in band intensity in small molecular components. The same result was observed when the temperature was set at 90 °C. The reason for the increase in small molecule bands was the hydrolysis of the gelatin subunit during heat treatment [26].

#### 3.1.2. FTIR of Tilapia Skin Gelatins

The FTIR of gelatins exhibited a similar characteristic spectrum and is presented in Figure 1B, albeit with slight variations. A distinct absorption signal at 1660–1650 cm^−1^ was observed, which can be attributed to the presence of Amide I and C=O bending. Furthermore, Amide A, Amide II, and Amide III appeared at around 3400–3410 cm^−1^ (N–H stretching), 1545–1555 cm^−1^ (N–H deformation), and 1235–1245 cm^−1^ (C–N stretching) [27]. The presence of amide III indicated the presence of a disordered structure in gelatin molecules, which may be associated with the absence of the triple helix structure. As the heat treatment time extended at a constant temperature, a slight red shift was observed in amide I, suggesting that heat treatment changed the structure of gelatin molecules and diminished the formation of intramolecular or intermolecular hydrogen bonds [28].

#### 3.1.3. Intrinsic Fluorescence of Tilapia Skin Gelatins

Intrinsic fluorescence spectra can be used to investigate the alterations in protein tertiary structure [29]. The exposure or hidden chromophores, such as tryptophan residues, may improve or diminish the intrinsic fluorescence intensity of proteins. The effect of heat treatment on the intrinsic fluorescence emission spectra of gelatins is depicted in Figure 1C. The wavelength peaks were observed unchanged in all gelatins. The intrinsic fluorescence intensity was decreased as the heat temperature increased, while the fluorescence intensity initially increased and then decreased with the extension of heating time for the same temperature (Figure S1). The results implied that heat treatment changes the molecular composition of gelatins, which was beneficial or unfavorable to the exposure of chromophore groups. Zhang et al. investigated the impact of heat treatment on the physical and chemical properties of Pacific oyster protein, revealing that fluorescence intensity initially increased and subsequently declined with the alterations in protein molecular composition [30]. This was consistent with the results of this study.

#### 3.1.4. Surface Hydrophobicity (*H*_0_) of Tilapia Skin Gelatins

*H*_0_ revealed the content of hydrophobic amino acid residues after the exposure of protein side chains. Studies indicated that *H*_0_ was associated with intrinsic fluorescence, emulsification, and various other properties [31]. The influence of heat treatment intensity on the *H_0_* of gelatins is displayed in Figure 1D. In comparison to gelatins heated for 2 h, *H*_0_ was rapidly increased after being treated for 5 h but then showed a noticeable decrease when the heat treatment time was 8 h. Furthermore, the *H*_0_ of gelatins was reduced as the extraction temperature rose. The correlation analysis (Figure S2) indicated that *H*_0_ exhibited a positive relationship with the range of fluorescence intensity (*p* < 0.05). The changes in *H*_0_ can be attributed to the alteration of molecular composition and the folding of proteins during heat treatment, which led to the exposure or hidden of the hydrophobic residues [32]. Similar phenomena were seen in prior investigations regarding the impact of ultrasound on the protein characteristics of oysters and the effect of heat treatment on functional characteristics of quinoa protein isolate [33,34].

#### 3.1.5. Emulsifying Properties of Tilapia Skin Gelatins

The EAI could assess the capability of protein to be absorbed at the interface between the oil and water phase during emulsion formation, while the ESI indicated the ability of protein to remain at the water–oil interface [35]. As shown in Figure 1E,F, the ESI changes in gelatin under different heat treatment intensities exhibited a similar trend to the EAI. For the same temperature, the EAI of gelatin heated for 5 h was significantly higher compared to 2 h and 8 h. This phenomenon can be attributed to the moderate hydrolysis of protein molecules under 5 h at 50 °C, 70 °C, and 90 °C, which enhancement the flexibility, and the surface-to-volume ratio of tilapia skin gelatin [36]. The proportion of protein molecules that participate in the formation of the interfacial layer was larger. In addition, a correlation study indicated a positive relationship between *H*_0_ and the EAI (*p* < 0.05). The higher *H*_0_ and emulsification indicate that gelatin molecules were easily adsorbed at the oil–water interface, hence improving the stability of HIPEs, which was advantageous for the storage and gradual release of fat-soluble flavor components.

### 3.2. Texture Properties of Tilapia Skin Gelatin Hydrogels

**Figure 2:**
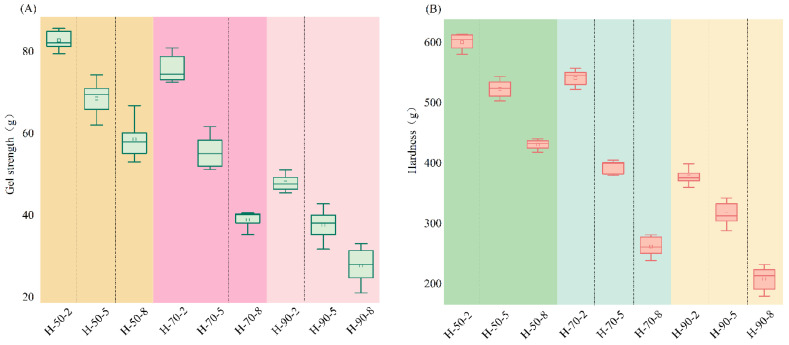
Gel strength (**A**) and hardness (**B**) of hydrogels stabilized by gelatins extracted at different temperatures and times.

Gel strength was considered a vital physical property of hydrogels, and the TPA test possesses a strong association with the sensory perception of textural characteristics [6]. Figure 2 shows that the gel strength and hardness of gelatin hydrogels exhibited a decrease with the increase in heating temperature and time, and the correlation analysis shown in Figure S2 revealed that both gel strength and hardness showed a negative association with heating temperature (*p* < 0.05). Previous studies indicated that the texture properties of gelatin hydrogels can be influenced by the chain length and conformation of the proteins [37]. The reduced gel strength and hardness may be attributed to the cleavage of hydrogen bonds and insufficient unfolding during gel network formation [38]. Table S1 indicates that the stickiness decreases as the heat treatment intensity increases. This observation may be due to the slight melting of hydrogels with lower gel strength, causing them to adhere to the probe surface and consequently increasing stickiness. Gelatins with varying gel strengths could regulate the rheological properties of gelatin emulsions, influencing their utilization in 3D printing. The gel strength of tilapia skin gelatins (25.22–79.94 g) were slightly inferior to Atlantic salmon skin gelatin (40–110 g) [39] and significantly lower than mammalian sources, such as pig skin gelatin (183.55–717.36 g) [13] and cowhide gelatin (244.4 g) [40]. The hardness of heat-treated tilapia skin gelatin varies from 600.73 g to 208.38 g, in comparison to the hardness of tuna and chicken skin gelatin, which are 460 g and 490 g [27], respectively. The variation in gelatin source and extraction methods were primary reasons for their differing textural qualities.

### 3.3. Three-Dimensional Printing Products

**Figure 3:**
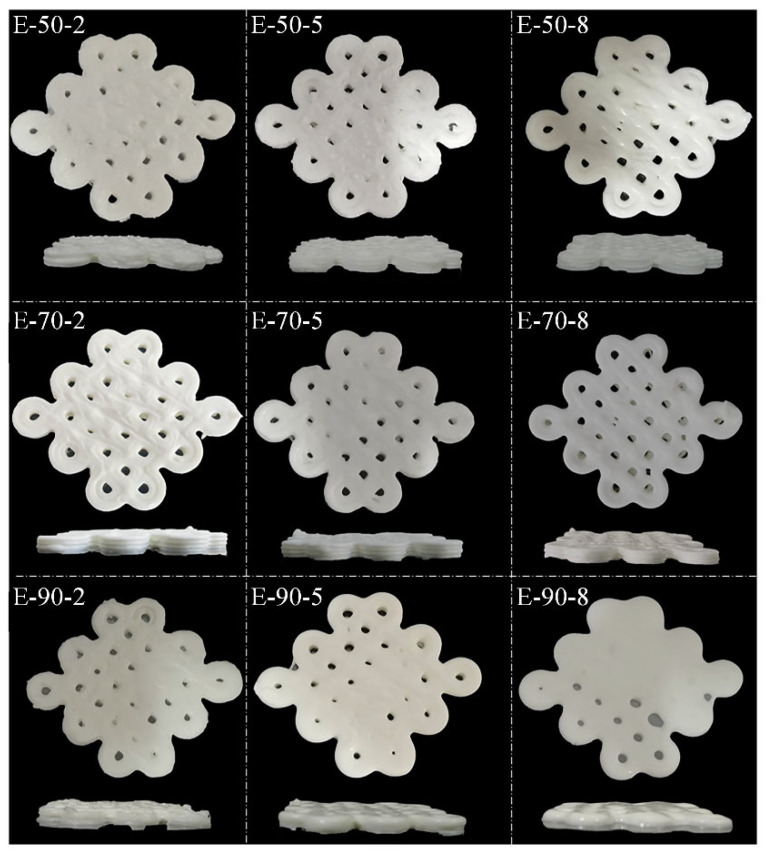
3D printing photographs of high internal phase emulsions (HIPEs) stabilized by gelatins extracted at different temperatures and times.

Figure 3 exhibits the photos of 3D printing shaped by HIPEs. All HIPEs can be extruded continuously. E-70-2, E-70-5 and E-70-8 showed well-defined boundaries and distinct stacking levels to reproduce the 3D model of Chinese knot. E-50-2 and E-50-5 showed rough edges and uneven surfaces, while E-90-5 and E-90-8 displayed indistinct textures post-printing. The incapacity of E-90-5 and E-90-8 to self-support may be attributed to the low gel strength of gelatin.

Previous studies have indicated that the characteristics of the printing ink play a crucial role in determining the outcome of 3D printing [7]. The HIPEs prepared by gelatins and soybean oil showed great potential as printing ink. The printed materials in this work can be utilized in food or nutrient delivery systems and food digital customization.

### 3.4. Rheological Properties of HIPEs

**Figure 4:**
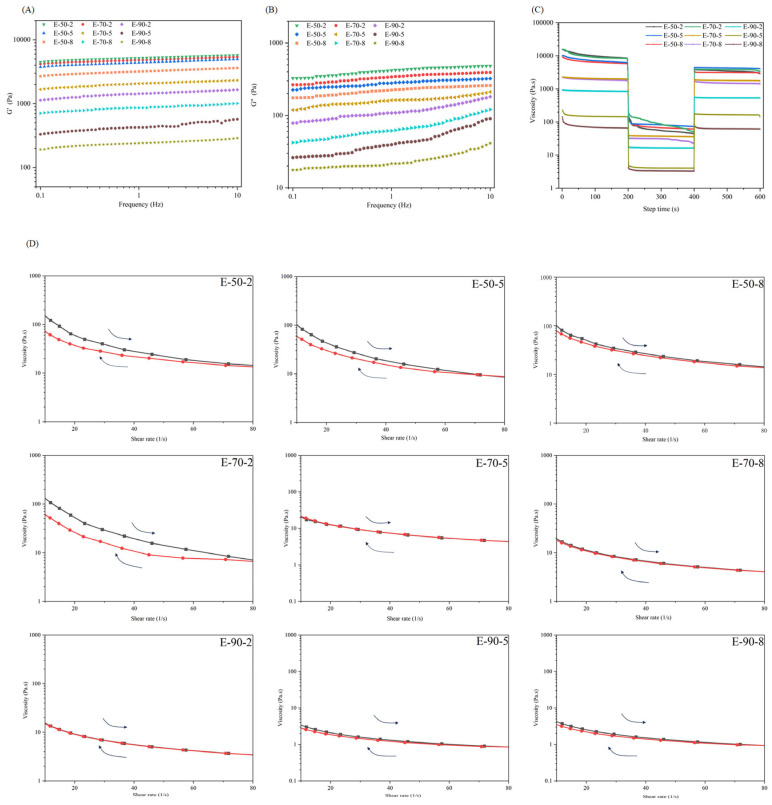
Rheological behavior of HIPEs stabilized by gelatins extracted at different temperatures and times. (**A**) Frequency sweep test of storage modulus (G′), (**B**) Frequency sweep test of loss modulus (G″), (**C**) 3ITT curves, (**D**), Cyclic shear ramp test.

Rheological behaviors of HIPEs were measured at a strain of 1%. Figure 4A,B demonstrates that G′ were larger than G″ throughout the whole range of frequencies, indicating the elasticity of HIPEs. The results were in line with the findings of casein emulsion [41]. Furthermore, E-50-2 exhibited the largest value of G′, whereas E-90-8 demonstrated the lowest value of G′. This result corresponded to the regulation of change in the gel strength of gelatin hydrogels.

3-ITT simulated the extrusion and shearing process during 3D printing. During the extrusion process, it was essential for HIPEs to possess sufficient and consistent fluidity during extrusion shearing. This allowed them to print on the substrate through the nozzle evenly. Subsequently, they must regain enough viscosity after printing to uphold the high definition and accuracy of the printed shape [42]. As shown in Figure 4C, all HIPEs exhibited a significant decline in viscosity during a transition of shear rate from 0.1 s^−1^ to 10 s^−1^. Conversely, when the shear rate returned to 0.1 s^−1^, the viscosity of the HIPEs promptly recovered. For the same temperature, the RR of HIPEs increased with the duration of gelatin heating. The *RR* of E-50-8, E-70-8, and E-90-8 were found to be 95.51%, 95.55%, and 98.33%, respectively (Figure S4), indicating a high level of *RR*. It was reported that a high viscosity recovery rate was advantageous to achieve precise printing structures [9].

Thixotropy reflected a decrease in apparent viscosity under shearing, followed by a gradual recovery when the shear reduced. A cyclic shear ramp flow, involving increasing and decreasing shear, was conducted to investigate the thixotropy of HIPEs. Figure 4D demonstrates the viscosity of HIPEs dropped as the speed increased, suggesting that HIPEs were easily extruded, while the viscosity of HIPEs gradually raised as the shear rate decreased in the subsequent process, indicating that HIPEs provided adequate support after extrusion [20]. Figure S5 reveals that E-50-2 and E-70-2 have a greater level of *Ti*. This could be attributed to their high initial viscosity and considerable structural rigidity. Furthermore, during the recycling procedure, the value of *Ri* for E-70-5, E-70-8, and E-90-2 exceeded 85% (Figure S6), which had a positive impact on the manufacturing of stable 3D-printed products [43].

### 3.5. Microstructure of HIPEs

**Figure 5:**
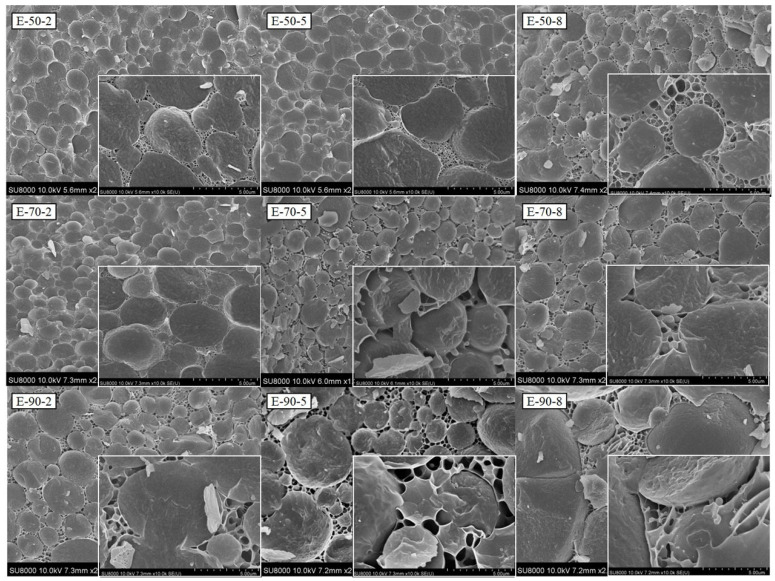
Microstructure of HIPEs stabilized by gelatins extracted at different temperatures and times.

The microstructure of HIPEs constructed by gelatin is shown in Figure 5. Gelatin generated a gel structure of networks surrounding oil droplets. As the intensity of heat treatment increases, the voids in the gel network structure enlarge. The oil droplets in E-50-2, E-50-5, and E-70-2 were enveloped by a denser gel network structure. In E-90-5 and E-90-8, the oil droplet sizes were inconsistent, and large voids existed within the network structure. The microstructure of HIPEs correlates with their texture and rheological characteristics. Previous studies indicated that gelatin with stronger gel strength may construct a more compact network structure [44]. The HIPEs developed in this study were O/W emulsions, indicating that the formation of a more compact gelatin network structure correlates with the higher G′ and viscosity of HIPEs.

## 4. Conclusions

The impact of gelatin with varying properties, derived from different heat treatment durations and temperatures, on the characteristics of HIPEs and the precision of 3D printing was assessed. The results indicated that gelatin undergoes a significant degradation after being heated at 70 °C for 5 h. The highest values of *H*_0_, fluorescence intensity, the ESI, and the EAI appeared when the heating time was 5 h in all temperatures. A correlation investigation indicated that *H*_0_ exhibited a positive correlation with fluorescence intensity, the EAI, and the ESI. Conversely, the heating temperature demonstrated a negative correlation with the gel strength and hardness of gelatin hydrogels. The gelatin treated at 70 °C for varying durations (2 h, 5 h, and 8 h) developed HIPEs appropriate for 3D printing. The variations in G′ and the viscosity of all HIPEs were mainly regulated by the gel strength of gelatins, and a denser network structure was formed in HIPEs with a higher G′. HIPEs suitable for 3D printing were constructed by gelatin prepared through a hydrothermal method in this work, which promotes the application in functional and personalized nutritious foods.

## Data Availability

The original contributions presented in this study are included in the article/Supplementary Materials; further inquiries can be directed to the corresponding author.

## Supplementary Materials

The following supporting information can be downloaded at: https://www.mdpi.com/article/10.3390/foods13244009/s1, Figure S1. Fluorescence intensity of gelatins from the skin of tilapia extracted at different temperatures and times; Figure S2. Correlation analysis of ESI, EAI, H0, gel strength, intrinsic fluorescence intensity, hardness, heating time and heating temperature of gelatins extracted at different temperatures and times; Figure S3. Shear strain sweep of HIPEs stabilized by gelatins from the skin of tilapia extracted at different temperatures and times; Figure S4. *RR* of HIPEs stabilized by gelatins from the skin of tilapia extracted at different temperatures and times during three-interval thixotropy test (3ITT); Figure S5. *Ti* of HIPEs stabilized by gelatins from the skin of tilapia extracted at different temperatures and times during during cyclic shear ramp test; Figure S6. *Ri* of HIPEs stabilized by gelatins from the skin of tilapia extracted at different temperatures and times during cyclic shear ramp test (*p* < 0.05); Table S1. Textural of hydrogels stabilized by gelatins from the skin of tilapia extracted at different temperatures for and times
