# Supplementary material for: Effect of Heat Treatment on Gelatin Properties and the Construction of High Internal Phase Emulsions for 3D Printing
Source: Foods. 2024 Dec 11;13(24):4009. doi: 10.3390/foods13244009 (PMC11728344; doi:10.3390/foods13244009)
Supplement: Supplementary file 1 [file foods-13-04009-s001.zip › foods-3311919-supplementary.pdf]

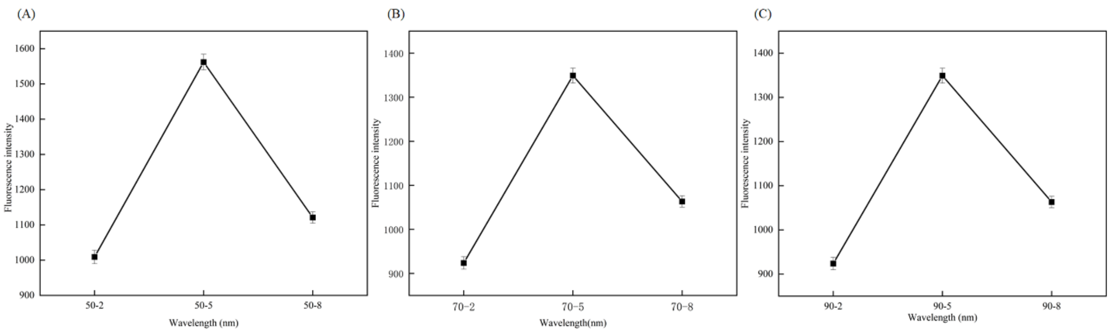

Figure S1. Fluorescence intensity of gelatins from the skin of tilapia extracted at different temperatures and times.

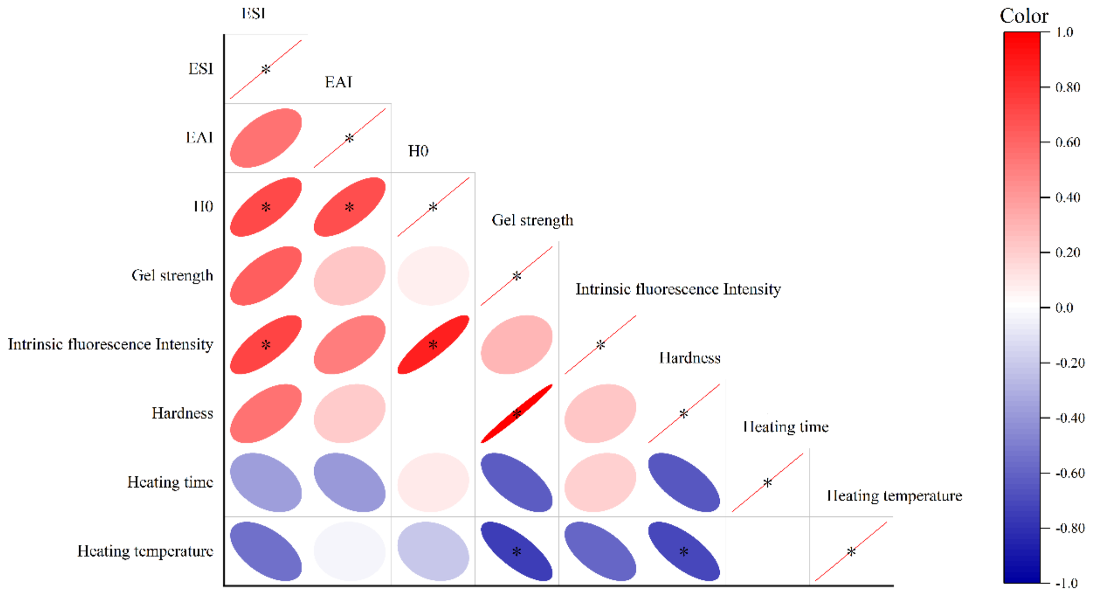

* p<=0.05

Figure S2. Correlation analysis of ESI, EAI, H_0, gel strength, intrinsic fluorescence intensity, hardness, heating time and heating temperature of gelatins extracted at different temperatures and times.

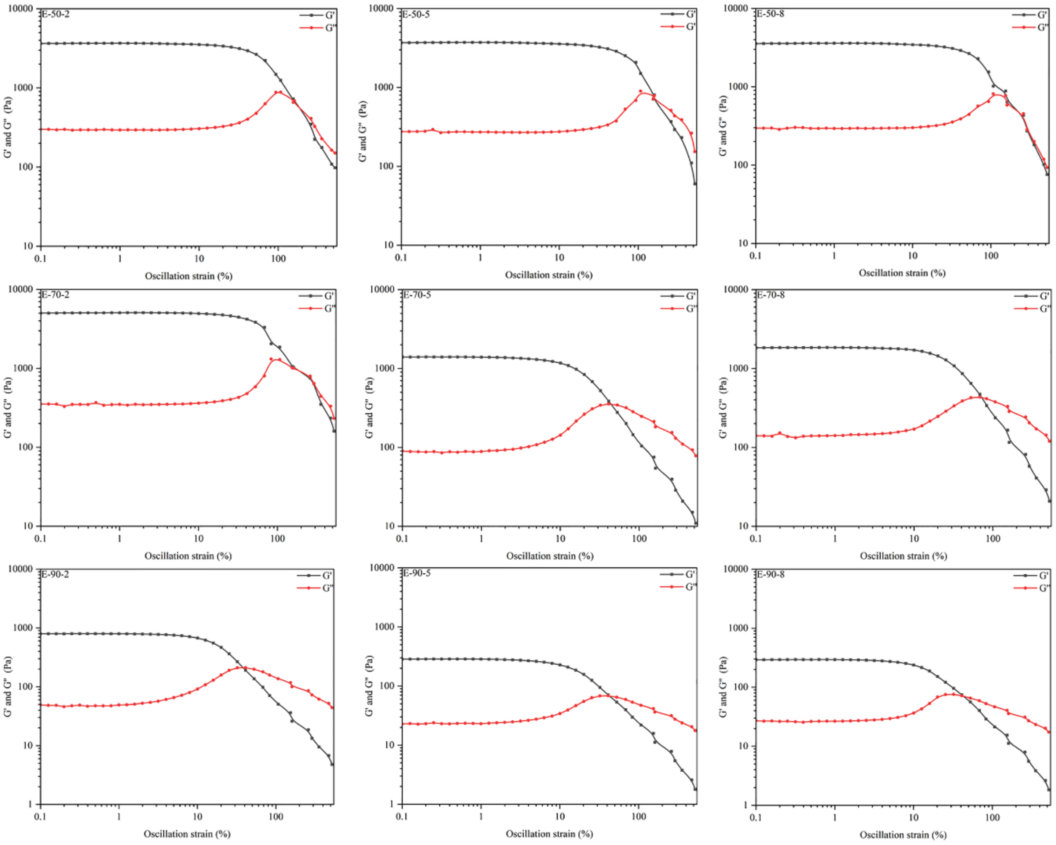

Figure S3. Shear strain sweep of HIPEs stabilized by gelatins from the skin of tilapia extracted at different temperatures and times.

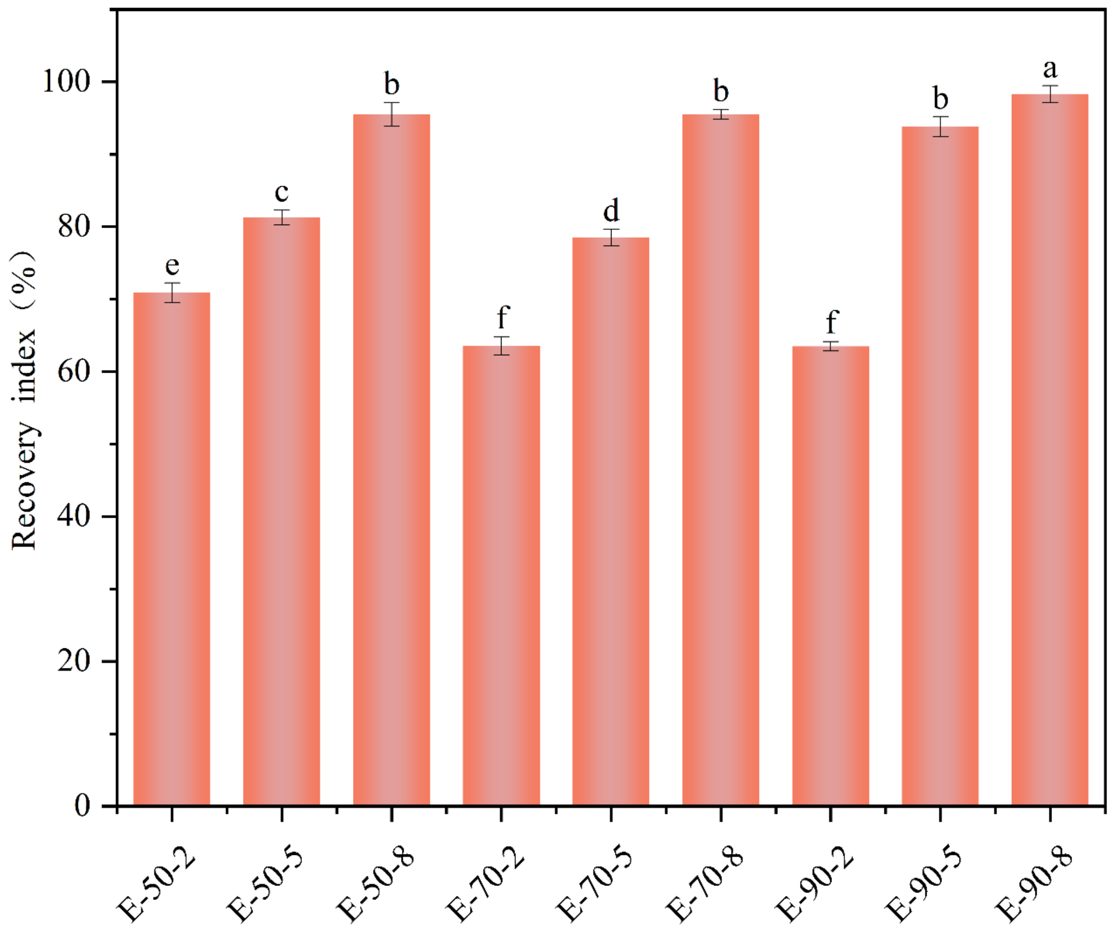

Figure S4. *RR* of HIPEs stabilized by gelatins from the skin of tilapia extracted at different temperatures and times during three- interval thixotropy test (3ITT). Note: Different letters indicate the significant difference between samples ($p<0.05$).

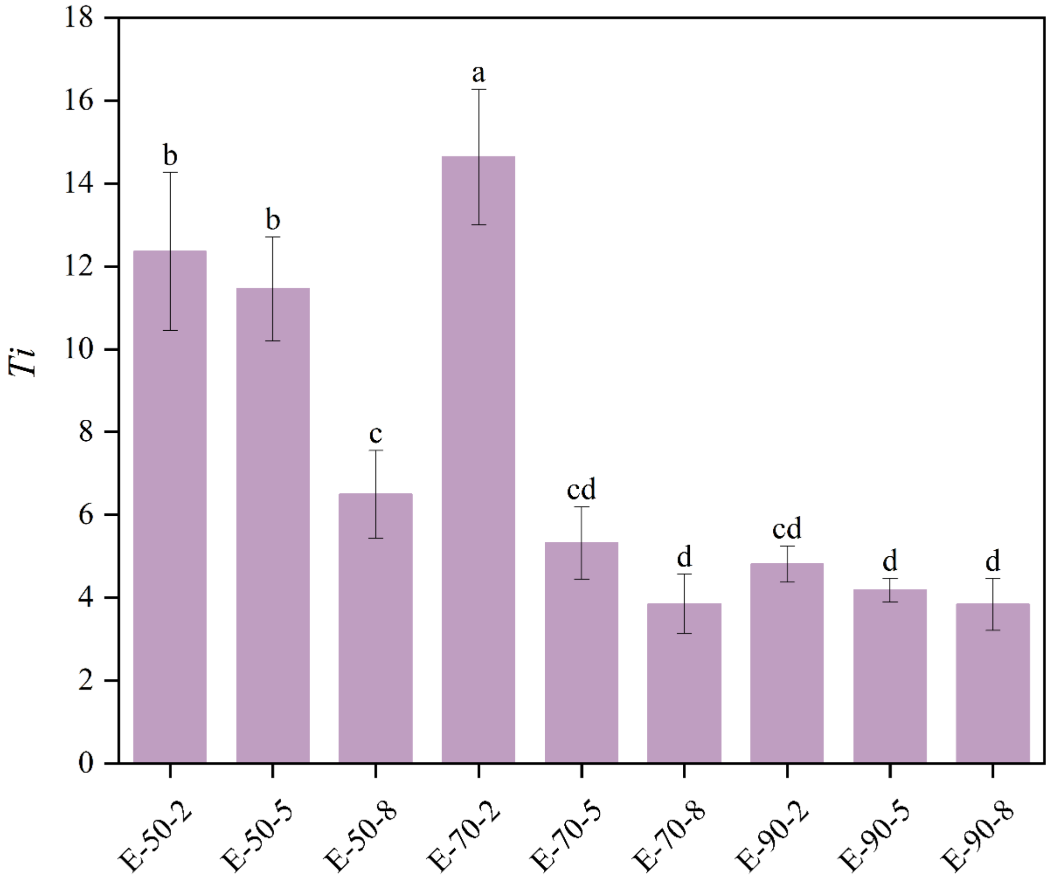

Figure S5. *Ti* of HIPEs stabilized by gelatins from the skin of tilapia extracted at different temperatures and times during during cyclic shear ramp test. Note: Different letters indicate the significant difference between samples ($p<0.05$).

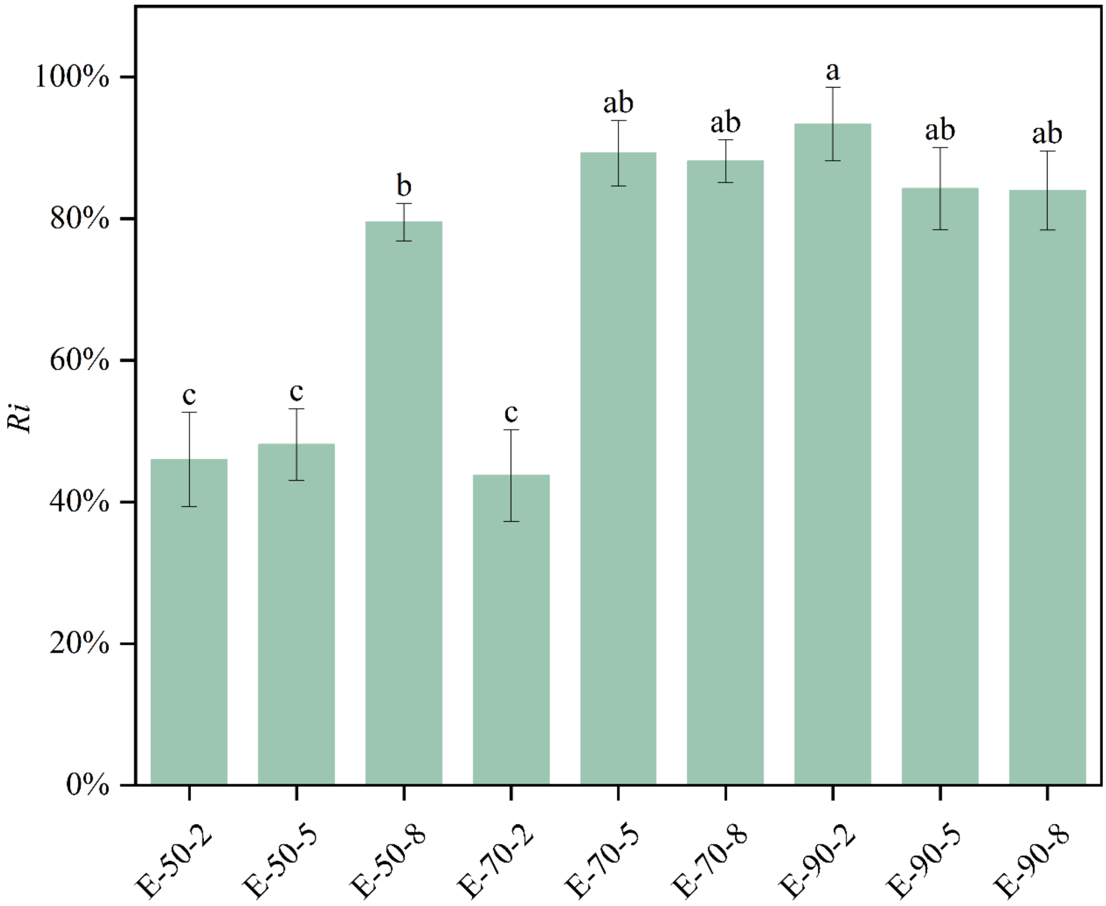

Figure S6. *Ri* of HIPEs stabilized by gelatins from the skin of tilapia extracted at different temperatures and times during cyclic shear ramp test ($p<0.05$).

Table S1. Textural of hydrogels stabilized by gelatins from the skin of tilapia extracted at different temperatures for and times.

Type	Stickiness (N)	Chewiness (N)
50-2	177.96 ± 12.12^{h}	519.56 ± 12.86^{a}
50-5	308.06 ± 16.09^{e}	435.97 ± 21.49^{c}
50-8	439.32 ± 13.02^{c}	387.42 ± 12.54^{d}
70-2	224.05 ± 9.60^{g}	477.28 ± 1.00^{b}
70-5	360.22 ± 10.67^{d}	333.23 ± 11.93^{e}
70-8	485.91 ± 8.73^{b}	215.81 ± 10.19^{f}
90-2	259.22 ± 13.01^{f}	333.01 ± 12.80^{e}
90-5	434.16 ± 7.02^{c}	236.58 ± 15.16^{f}
90-8	554.99 ± 15.19^{a}	155.77 ± 6.88^{g}
